# Transmission Electron Microscopy-based characterization of Extracellular Vesicles from plasma and serum from Parkinson´s Disease patients

**DOI:** 10.1186/s12964-025-02383-w

**Published:** 2025-09-18

**Authors:** Alexander Weiss, Alex Florin Meissner, Fanni Annamária Boros, Martin Regensburger, Regina Verena Taudte, Andreu Matamoros-Angles, Philipp Arnold, Friederike Zunke

**Affiliations:** 1 Department of Molecular Neurology, University Hospital Erlangen, Friedrich-Alexander-University Erlangen-Nürnberg (FAU), Erlangen, Germany; 2 Institute of Functional and Clinical Anatomy, Friedrich-Alexander-University Erlangen-Nürnberg, Erlangen, Germany; 3 Institute of Laboratory Medicine and Pathobiochemistry, Molecular Diagnostics, Philipps-University Marburg, Marburg, Germany; 4 Institute of Neuropathology, University Medical Center Hamburg-Eppendorf, Hamburg, Germany

**Keywords:** Extraccellular Vesicles, Parkinson's Disease, Transmission Electron Microscopy, Plasma, Serum

## Abstract

Parkinson’s disease is a neurodegenerative disorder with no curative treatment option and objective biomarker profile. Extracellular Vesicles (EVs) are membrane-enclosed biological nanoparticles released from all cells of the human body. In this pilot study we compare plasma- and serum-derived EVs from Parkinson’s disease (PD) patients and healthy controls (HC) utilizing a precipitation-based method. Additionally, we employ an L1CAM antibody to selectively enrich for L1CAM-positive EVs from the total plasma-/serum-derived EV fractions. Successful EV enrichment was shown in western blot experiments for CD63 and for L1CAM as well as in metabolomic analysis for a HC sample. In a side-by-side quantification,. which we based on transmission electron microscopic images from negative stain samples, we identify small but significant differences between EV diameter from PD patients and HC. To streamline the quantification process, we introduce an ImageJ-based computer algorithm for (semi-)automated quantification of EVs from negative stain electron micrographs. We observe that this (semi-)automated quantification determines a smaller diameter than manual quantification. However, the difference between PD and HC group is systematic and reveals the same relative differences calculated from manually measured total plasma-derived EV particles. In this pilot study, we introduce a new workflow implemented into an ImageJ plugin enabling to determine differences in EV size within TEM images. For our data set of plasma-derived EVs from PD patients and HC, we find small, yet consistent differences. We feel that this study contributes to the search of a clinical biomarker for PD.

**Supplementary Information:**

The online version contains supplementary material available at 10.1186/s12964-025-02383-w.

## Background

Extracellular Vesicles (EVs) are lipid bilayer-enclosed biological nano-particles naturally released by all cells of the human body. In recent years, their role in inter-cellular and inter-organ communication has become increasingly evident [1, 2]. They contain proteins, metabolites, and nucleotides that represent physiological and/or pathological processes ongoing in the mother cell [3–5]. Profound insights into these nanoscale vesicular entities have substantially augmented our understanding of intracellular biochemical processes. In numerous diseases, EVs have been identified as pathogenic descriptors, but also mediators, therapeutic targets, and innovative vectors for precision-medicine, thus underscoring their significant potential across an array of biomedical and translational medical applications [6–8].

With the increasing recognition of EVs as central mediators in a wide spectrum of physiological and pathological processes, methodologies for EV isolation from human-derived biofluids have undergone remarkable advancement over recent years. Currently, differential ultracentrifugation (UC) and size-exclusion chromatography (SEC) are regarded among the most effective and purity-enhancing techniques for EV extraction from biofluids (i.e., blood, urine, and cerebrospinal fluid (CSF)), enabling the production of highly enriched EV preparations, when protocols are carefully optimized [9–11]. However, these methods are often hampered by high costs, labour-intensive procedures, and limited yield of EV material, due to substantial loss of material occurring during the purification process [9, 12]. In the present study, we focus on a precipitation-based enrichment approach to isolate EVs from blood, which offers a streamlined, rapid alternative to conventional isolation techniques [12, 13], promoting EV enrichment followed by subsequent separation via low-speed centrifugation.

Characterization of nanoscale particles including EVs (in line with MISEV guidelines [9]), can be achieved through two well-established techniques: Transmission Electron Microscopy (TEM) and Nanoparticle Tracking Analysis (NTA) [9]. TEM affords high-resolution imaging of individual particles, enabling detailed analysis of particle morphology and interactions with antigenic targets [9, 14]. A shortage for TEM analysis is the labour-intensive image analysis to obtain a quantitative overview of the entire EV preparation. In contrast, NTA employs a light-scattering approach, enabling the detection and tracking of thousands of particles within a defined illumination volume to calculate particle concentration and size distribution, which generates statistically robust data, which may also introduce a lot of variability and lack of reproducibility depending on the equipment and protocol used for the analysis as previously described [9, 14–16]. Additionally, it is essential to analyse the biochemical composition of samples, as the presence of specific proteins – e.g. tetraspanins CD9, CD63, CD81 or the cytosolic Alix − serve as a critical markers to confirm the presence of EVs in human-derived samples [9].

Parkinson´s Disease (PD), is a neurodegenerative disorder characterized by the pathological accumulation of misfolded alpha-synuclein (a-syn) in the brain, which leads to the damage and loss of dopaminergic neurons and consequently manifests in extrapyramidal motor symptoms [17–19]. EV research represents a significant opportunity for transformative advancements in the description/monitoring of PD pathophysiology [20]. While clinical diagnostic accuracy of the Movement Disorders Society criteria for PD is sufficient in the motor manifest disease stages [21, 22], additional biomarkers are urgently needed to make a biological and objective diagnosis during the pre-symptomatic and prodromal phase. Recent studies have proposed a role for EVs in the pathological spread of disease markers associated with PD, potentially accelerating disease progression [23–26]. Stratifying for specific EV populations, like L1CAM-positive EVs (L1CAM-EVs) could represent a notable advancement in clinical research [27–33]. Quantification of disease-associated proteins within L1CAM- EVs has demonstrated considerable promise in distinguishing PD patients from healthy controls (HC) and in facilitating differential diagnosis [20, 28, 29, 34–36]. Furthermore, a systematic review of recent studies has underscored the potential of serum-derived L1CAM-EVs as a screening tool to identify prodromal PD patients in cohorts with an increased PD risk [37].

In this study, we analyze EV preparations isolated from the blood plasma and serum of PD patients and healthy controls (HC) using a simple precipitation method, and compare their morphology and size using TEM analysis. We additionally employ L1CAM-targeted immunoprecipitation to enrich for a L1CAM-positive EV subpopulation.

Notably, we have implemented a new plugin running under ImageJ (*EV finder*), facilitating efficient automatic or semi-automatic measurement of EV dimensions from TEM images. A side-by-side comparison of a manually and computer script assisted TEM data set demonstrates the applicability in automated and semi-automated mode. Application of this ImageJ implemented script reduces the time for TEM image analysis to a minimum (minutes instead of hours or days), increases objectivity and transforms TEM images of EV into easily quantifiable data. Our pilot study reports a workflow that will be utilized for follow-up studies with larger cohorts at different sites or across cohorts with different disease onset times.

## Material & Methods

### Sample Collection

Human blood samples were collected in the Department of Molecular Neurology, University Hospital Erlangen (*Erlangen, Germany*). We recruited individuals with Parkinson´s disease (PD) (*n* = 5) and community derived healthy control persons (HC) without clinical signs of a neurological disease (*n* = 5), matched for age and sex. All donors provided written informed consent (protocol n° 17–259_3-B, ethics committee of the Friedrich-Alexander-Universität Erlangen-Nürnberg). Suppl. Figure 1 A provides an overview of the baseline characteristics of our cohort. After collection of plasma in Lithium-Heparin (LH) tubes *(Sarstedt*,* Nümbrecht*,* Germany*,* #02.1065)* and serum in Serum CAT tubes *(Sarstedt*,* Nümbrecht*,* Germany*,* #01.1601)*, we separated plasma and serum from cellular debris by centrifugation (HERAEUS Multifuge 1 S-R; Swing-out rotor Sorvall 75902000; rotor brake on max) at 2,500 × g for 10 min after transport to the laboratory (5–10 min) followed by further centrifugation at 2,000 × g for 20 min and 10,000 × g for 20 min at room temperature (RT). Subsequently, samples were stored at −80 °C for up to several month.

### Isolation of Extracellular Vesicles (EVs)

Prior to EV isolation serum or plasma, samples were thawed on ice. EVs were separated from plasma using the ThermoFisher Thermo Fisher´s Total Exosome Isolation Kit (TEIK) *(ThermoFisher Scientific Inc.*,* Waltham*,* MA*,* United States*,* #4484450)* and the Total Exosome Isolation Reagent (TEIR) *(ThermoFisher Scientific Inc.*,* Waltham*,* MA*,* United States*,* #4478360)* for serum samples, following the standard protocol from the manufacturer [38]. Proteinase K (PK) treatment was omitted to protect Surface proteins on EVs. The following procedure for plasma is described in parallel to the serum protocol, differing only by the according exosome precipitation reagent. A total of 500 µl of plasma, diluted with 250 µl of Dulbecco’s Phosphate-Buffered Saline (DPBS) *(Sigma-Aldrich*,* St. Louis*,* MO*,* United States*,* #D8537-500ML)*, was mixed with 150 µl of exosome precipitation reagent. The mixture was incubated for 10 min at RT, followed by centrifugation (Eppendorf Centrifuge 5430 R with a fixed-angle rotor Eppendorf FA-45-24-11-HS pre-cooled to 4 °C) at 10,000 × g for 5 min at 4 °C to isolate EVs from the plasma. After an additional 30-second centrifugation at 10,000 × g and 4 °C, the Supernatant was discarded. The EV pellet was then resuspended in DPBS. For TEM imaging, 0.9% NaCl was used for resuspension. For western blot analysis samples were stored at −20 °C for 1–2 weeks and for TEM analysis at 4 °C for 1–3 days. Suppl. Figure 1B provides an overview of the EV isolation process.

### Immunoprecipitation of L1CAM-positive EVs

L1CAM-positive EVs, in the following denoted as L1CAM-EVs were separated from the total EV pool by immunoprecipitation, schematically illustrated in Suppl. Figure 1 C. After EV enrichment, the pellet was resuspended in 300 µl DPBS and incubated with 2 µg of anti-L1CAM C2 mouse antibody (*Santa Cruz Biotechnology*, *Dallas*,*TX*,* United States*,* #sc-551430*; Suppl. Table 1) at 4 °C overnight. Protein A/G-agarose beads *(Santa Cruz Biotechnology*,* Dallas*,* TX*,* United States*,* #sc-2003)* were blocked in 3% bovine serum albumin (BSA). 20 µg of blocked beads were added to the EV samples and incubated for four hours at 4 °C. Washing with DPBS and Subsequent centrifugation for 5 min at 1,000 × g was performed to generate a pellet containing the L1CAM-EVs.

### Transmission Electron Microscopy (TEM) and Nanoparticle Tracking Analysis (NTA)

Negative stain TEM was performed as previously described [39–41]. 3 µl of EV sample was applied to a freshly negative glow discharged (25 mA for 15 s) continuous carbon 300 mesh copper grid (*Science Service Munich*,* Munich*,* Germany*,* #ECF300-Cu-50*) placed into an inverse pair of forceps. The sample was immediately washed twice with 5 µl of 1% aqueous uranyl acetate (UA) solution (*Science Service Munich*,* Munich*,* Germany*,* #E22400*). For this, the edge of the grid facing away from the forceps was touching a filter paper (Soft tissue), which blotted of the sample. Immediately, the first 5 µl of UA solution were added at the side of the forceps, resulting in a flow over the grid and immediate blotting into the filter paper. Washing was repeated and then the edge between forceps and grid was blotted against filter paper to reduce sticking of the grid to the forceps. The grid was then allowed to air-dry in the inverse forceps at room temperature for at least two minutes prior to imaging in the TEM. TEM images were taken on a JEOL 1400Plus TEM *(JEOL Germany*,* Munich*,* Germany)* at 120 kV and nominal magnifications of 30,000x and 50,000x. TEM pictures were analysed with ImageJ software *(National Institutes of Health (NIH)*,* Bethesda*,* MD*,* United States)*. All TEM images were acquired by the same operator at different positions of the grid. Changing grid position helps to obtain a more unified overview on the sample and omits spot-specific staining alterations.

Nanoparticle tracking analysis (NTA) was conducted in cooperation with the Chair of Pharmaceutical Biology, Department of Biology *(FAU Erlangen-Nürnberg*,* Germany)*, following established protocols [42]. We used ZetaView^R^ Z-NTA TWIN PMX-220–12 F-R5 system *(Particle Metrix*,* Meerbusch*,* Germany)*, which is designed to capture the Brownian motion of nanoparticles via video and further calculate the particle size by analysing the diffusional movements of the particles.

### ImageJ implemented script EV-TEM Analyzer

To develop our ImageJ implementable plugin, the *EV finder*, we utilized image processing tools available and connected them with the ImageJ implemented macro language. Our script runs on ImageJ version v1.54d (and in earlier versions) and on FIJI. Following copying it into the ImageJ *plugins* folder and restarting ImageJ/FIJI, it can be accessed through the *plugins* drop down menu. Several GUI (graphical user interface) will open and ask for input values. Our documentation guides the user through all input values and we provide extended documentation on how to determine these input values for individual data sets in case default values do not work. *EV finder* can be requested from us free of charge and will be available upon publication on our homepage (www.evi.forschung.fau.de). When using *EV finder* we ask you to cite this paper.

### Biochemical analysis methods

For Sodium dodecyl sulphate polyacrylamide gel electrophoresis (SDS-PAGE) and Western Blotting, EV samples were lysed in 100 µL Triton buffer *(1% Triton-X100*,* 10% glycerol*,* 150 mM NaCl*,* 25 mM HEPES at pH 7.4*,* 1 mM EDTA*,* 1.5 mM MgCl*_*2*_*)* containing 1 × protease inhibitor cocktail *(Roche*,* Penzberg*,* Germany*,* #11836145001)*, 50 mM NaF, 2 mM NaVO_4_, 1 mM PMSF by incubating the samples for 30 min on ice, and Subsequently centrifuging at 2,100 × g for 5 min.

The protein amount of the samples was determined with bicinchoninic acid (BCA) assay, using Pierce™ BCA Protein Assay Kit *(ThermoFisher Scientific Inc.*,* Waltham*,* MA*,* United States*,* #23225)*. SDS-PAGE was conducted with a Tris-glycine buffered system, consisting of SureCast™ resolving buffer and stacking buffer *(ThermoFisher Scientific Inc.*,* Waltham*,* MA*,* United States*,* #HC2215)*. Samples of 20 µL to 25 µL containing 15 µg to 40 µg of total protein were boiled with 5 × Laemmli buffer *(0.3 M Tris-HCl*,* pH 6.8*,* 10% SDS*,* 50% glycerol*,* 5% ß-mercaptoethanol*,* 5% bromophenol blue)* for 5 min at 95 °C, with Subsequent loading on 10% or 12% Tris-glycin gels and Subjected for a duration of 1.5 h to electrophoresis with Tris-glycin SDS buffer *(25 mM Tris*,* 192 mM glycine*,* 1% SDS)*. Separated proteins were transferred onto polyvinylidene fluoride (PVDF) membranes. Primary antibodies (**suppl. Table 1**) diluted 1:500 or 1:1,000 in TBS Intercept antibody diluent *(LI-COR Biosciences*,* Lincoln*,* NE*,* United States*,* #927-60001)* were added to the membranes and incubated overnight at 4 °C. The following day the primary antibody solutions were replaced with secondary antibodies diluted 1:10,000 and the membranes were incubated for 1 h at room temperature. Signals were detected using an Odyssey 9120 system *(LI-COR Biosciences*,* Lincoln*,* NE*,* United States)*, data were quantified with ImageStudioLite 5.2 *(LI-COR Biosciences*,* Lincoln*,* NE*,* United States)*. Acrylamide gels were stained with Coomassie Brilliant Blue (CBB) staining solution *(0.02% CBB G-250 (Carl Roth*,* Karlsruhe*,* Germany*,* #9598.1)*,* 5% aluminium sulphate-(14–18)-hydrate*,* 10% ethanol (96%)*,* 2% orthophosphoric acid (85%))* to provide total protein staining.

In addition to Western blot analysis, we performed untargeted metabolomics analyses in cooperation with the Institute of Experimental and Clinical Pharmacology and Toxicology in Erlangen *(FAU Erlangen-Nürnberg*,* Germany)*. For this, 500 µg of EV samples were lysed in 1 ml 1 mL lysis buffer (containing *80% HPLC grade methanol in LC-MS grade water*,* 0.1 mM tridecanoic acid*,* 0.015 mM DL-2-fluorophenylglycine*,* 0.06 mM d6-cholesterol*,* and 0.05 mM DL-4-chlorophenylalanine05 mM)*. Metabolomic analyses were conducted utilizing Orbitrap-MS Q Exactive Focus *(ThermoFisher Scientific Inc.*,* Waltham*,* MA*,* United States)* to detect compounds. Resulting raw files were processed with Compound Discoverer 3.1 software *(ThermoFisher Scientific Inc.*,* Waltham*,* MA*,* United States)*, further comparing the results with various online databases, such as mzCloud™ *(HighChem LLC*,* Bratislava*,* Slovakia)*, mzVault 2.1 *(ThermoFisher Scientific Inc.*,* Waltham*,* MA*,* United States)* and the Human Metabolome Database (HMDB) [43].

### Statistics

Statistical analyses were performed utilizing GraphPad Prism Version 10 (*GraphPad Software Inc.*,* San Diego*,* CA*,* United States*). The utilized test to determine statistical significance is provided in each figure legend, and p-values are denoted in each chart. Statistical significance was considered at *p* ≤ 0.05.

## Results

### Biochemical characterization of EV profiles

In accordance with MISEV guidelines of the International Society of EVs (ISEV) [9], we enriched and evaluated total EVs and L1CAM-precipitated EVs from a small PD patient and HC cohort (Suppl. Figure 1 A-C). Beyond employing biophysical approaches for particle quantification, as detailed in the subsequent section, we analysed the biochemical composition of total EV and L1CAM-EV samples.

In WB analysis, we successfully demonstrate the presence of EV markers in our total EV and L1CAM-positive EV population (Suppl. Figure 2 A). The tetraspanin CD63 (EV marker) was present in both, total EVs and L1CAM-EVs, with no statistical significant difference (*p* = 0.0578) between the groups. Conversely, L1CAM exhibited significantly higher levels in L1CAM-EVs compared to the total EV pool (*p* = 0.0461). Interestingly, despite loading the samples based on the same total protein amount measured by BCA assay, CBB signals revealed a significant increase of measured total protein in EVs (*p* = 0.0062), compared to L1CAM-EVs (Suppl. Figure 2 A).

In addition to the WB analysis of EV surface markers, we analysed the metabolic composition of our EV samples via untargeted metabolomic analysis (Suppl. Figure 2B). We assessed technical triplicates of one HC plasma-derived EV sample, leading to the identification of 77 metabolic compounds across all three samples. The majority (63.3%) of all detected compounds were classified as “lipids and lipid-like molecules”, with glycerophospholipids accounting for 67.4% of lipids identified within this group. The second largest class of identified compounds were “organic acids and derivatives”, comprising 18.2% of the total metabolites, mostly consisting of amino acids, peptides and analogues being in line with published data for EV composition [44, 45].

### Quantitative insights into particles isolated from plasma and serum via transmission electron microscopy

**Fig. 1:**
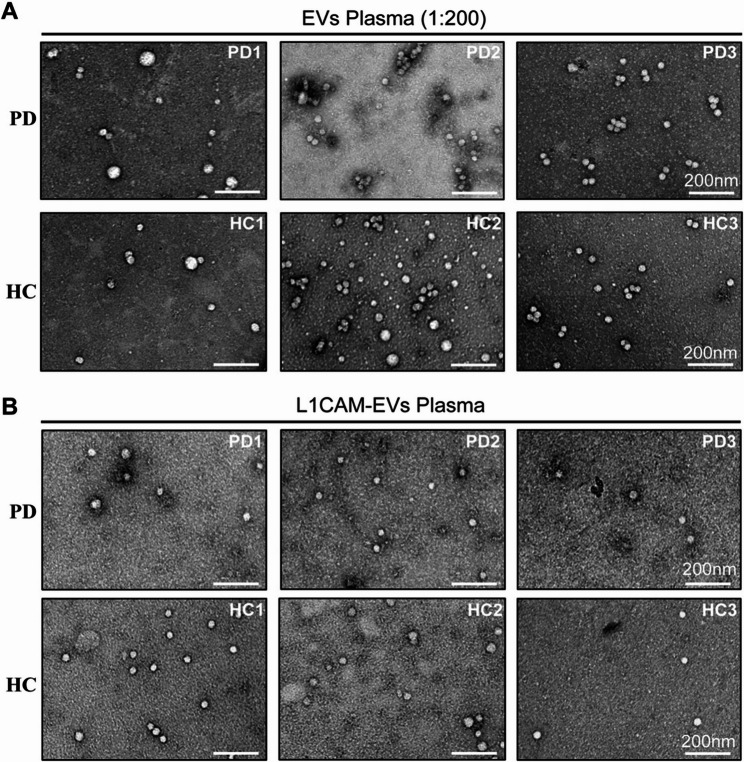
TEM images of plasma EVs and L1CAM-positive plasma EVs. **A** Representative TEM images of plasma-derived EVs from three individuals of the PD group (PD1 – PD3) and three individuals of the HC group (HC1 – HC3), diluted 1:200 in 0.9% NaCl. **B** Representative TEM images of plasma-derived L1CAM-EVs from three individuals, similar to A, but without further dilution. Scale bar = 200 nm

Utilizing TEM imaging, we evaluated the appearance of particles isolated from plasma samples of PD patients and HC (Fig. 1A, B). Predominantly, these particles displayed small, near-spherical morphologies with diameters ranging between 20 nm and 80 nm. The overall morphology and structural characteristics appeared consistent across all experimental groups (PD vs. HC; plasma vs. serum). The general appearance and morphology of the particles in TEM imaging were observed to be consistently similar across all groups.

In contrast to the undiluted L1CAM-EVs, the total EV-containing samples underwent a 1:100 to 1:200 dilution prior to TEM analysis. Despite this substantial dilution, the particle density within the visualized frames remained largely comparable. This preservation of density highlights the effectiveness of the immunoprecipitation approach, which significantly reduced overall particle count while selectively enriching target-specific vesicles.

Comparisons between plasma-derived and serum-derived particles via TEM demonstrated comparable images, in overall quality, particle count and particle size (Suppl. Figure 3 A, B). However, the particle density, quantified as the number of particles per defined frame, was significantly higher in serum-derived samples for both total EVs (*p* = 0.0198) and L1CAM-Evs (*p* = 0.0406) in comparison to plasma-derived EV samples (Supp. Figure 3C). Interestingly, TEM imaging provided compelling visual evidence of anti-L1CAM antibody interaction with L1CAM-EVs (Suppl. Figure 3D). A distinct 15 nm stripe-like structure was identified in association with particles, likely representing antibody binding to its target.

**Fig. 2:**
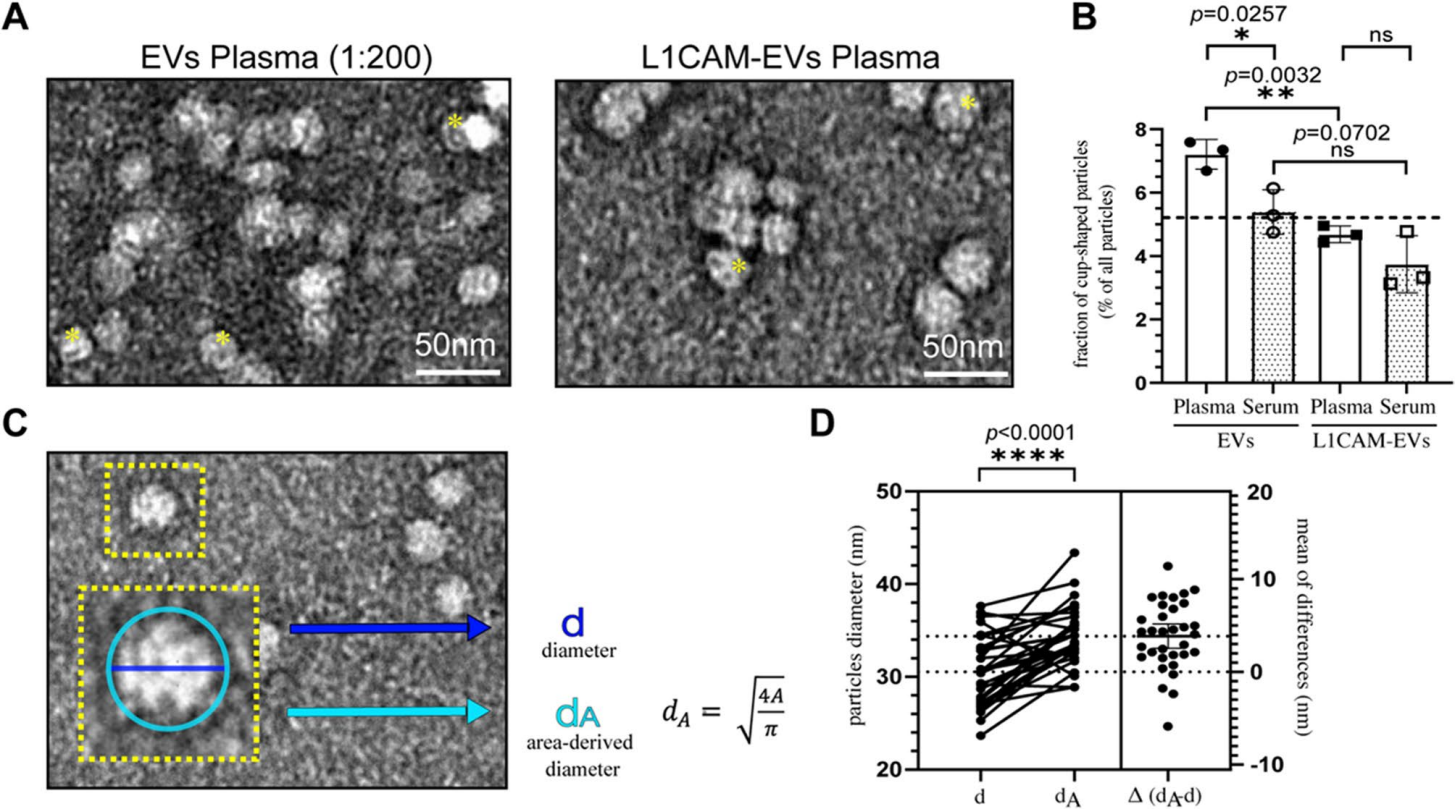
Morphology and Size of EVs in TEM imaging. **A** Two representative TEM images of plasma-derived EVs and L1CAM-EVs from one HC individual, EVs are further diluted 1:200 in 0.9% NaCl. Yellow stars (*) mark particles, which exhibit “cup-shaped” morphology. Scale bar = 50 nm. **B** Fraction of “cup-shaped” particles in relation to all detected particles, comparing plasma- and serum-derived EVs as well as L1CAM-EVs, *n* = 3 images of one HC individual. Error bars provide mean values ± SD; unpaired t-test with Welsh´s correction was performed; ns = not significant with *p* ≥ 0.05; */** = significant with *p* < 0.05. **C** Schematic comparison of two approaches to determine the diameter of particles images via TEM: dark blue approach represents direct diameter (d) from one edge to the opposite edge, light blue approach represents area-derived diameter (dA), by calculating the surface area of one particle and calculating the derived diameter with the illustrated formula. **D** Comparison of *n* = 33 particle diameters, imaged via TEM in one HC individual, measured by both approaches as explained in C. Paired t-test was performed; **** = statistically significant with *p* < 0.0001

Consistent with previous studies [9, 46, 47], characteristic “cup-shaped” morphology of EVs was observed upon negative staining TEM (Fig. 2A). This morphology, attributed to dehydration-induced artifacts during fixation [9, 14], was present in 3–8% of the particles across different samples. The frequency of cup-shaped particles was significantly higher in EVs compared to L1CAM-EVs in plasma-derived samples (*p* = 0.0032) and a trend towards cup-shaped EVs was observed in serum-derived samples (*p* = 0.0702). Additionally, plasma-derived particles exhibited a greater proportion of cup-shaped structures compared to serum-derived particles, observed in both EVs (*p* = 0.0257) and L1CAM-EVs (*p* = 0.2044, Fig. 2B).

### Quantitative analysis of particle diameters: a comparative evaluation of tem measurements

**Fig. 3:**
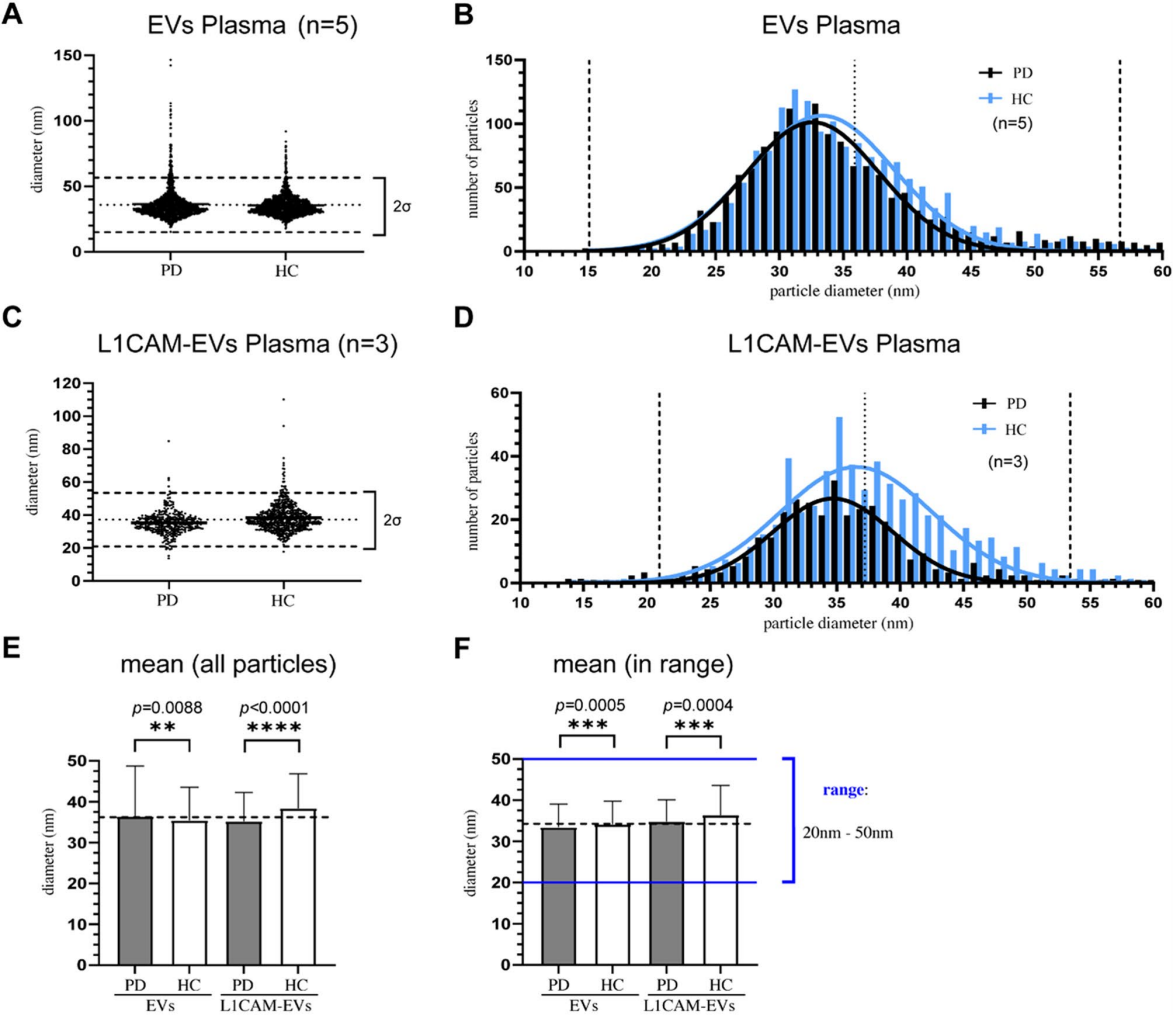
Size distribution in TEM (PD vs. HC). A + B. Comparison of particle diameters of plasma-derived EVs from *n* = 5 PD individuals and *n* = 5 HC individuals, determined via TEM analysis. **A **overview over all detected particles **(B)** presents a frequency distribution in mainly the 2SD interval. **C + D** Comparison of particle diameters of plasma-derived L1CAM-EVs from *n* = 3 PD individuals and *n* = 3 HC individuals, determined via TEM analysis, similar to EV-analysis above. **A-D** Dotted Lines represent mean values of all particles shown in the corresponding diagram; dashed Lines represent upper and lower Limits of the 2SD interval of all particles, representing the range in which 95.4% of all detected particles were observed. **E** Diagram of mean values (± SD) of all detected particles, comparing EVs in PD and HC group with L1CAM-EVs in PD and HC group. **F** Comparison of similar groups of **E**, but only comparing particles in the range between 20 nm and 50 nm. **E + F **Dashed line represent mean value over all four groups. Unpaired t-test was performed to determine the statistical significance, p-values are provided in each graph

The estimation of EV particle diameters differs depending on the method applied. Comparing NTA and TEM results in a larger particle diameter measured for NTA due to the presence of the hydration shell [15, 48, 49]. To analyse EV diameter from TEM images, two basic principles can be applied: The first approach is the direct linear measurement across the maximal distance between two opposing edges of a particle (Fig. 2C, dark blue line: linear diameter). The second approach calculated the particle diameters (*d*_*A*_) from the projected surface area (*A*) of the particles, assuming ideal spherical geometry, and applying the formula: *d*_*A*_=√(4*A*/π) [14] (Fig. 2C, light blue circle: area-based diameter). Both methods, however, are subject to limitations, as not all particles appear as perfect spheres in TEM imaging, with some present cup-shaped deformities. To assess the reliability of these methods, we conducted a detailed analysis of *n* = 33 particles visualized in TEM images of plasma-derived EVs from a control individual (Fig. 3D). The results demonstrate that area-derived calculated diameters yielded significantly larger diameters compared to diameters determined by linear measurements (*p* < 0.0001). On average, area-derived diameters exceeded line-based diameters by *d* = 3.87 nm, representing an 11.25% increase (Fig. 3D). Based on these findings, we selected the linear measurement approach as the standard for all subsequent manual diameter analyses.

Interestingly, we observed significant differences in the mean particle diameter when comparing plasma-derived particles to serum-derived particles for both total EVs and L1CAM-EVs. Serum-derived particles were consistently and significantly smaller than their plasma-derived counterparts (*p* ≤ 0.0001) (Suppl. Figure 3B). To complement TEM-based diameter measurements, NTA was employed for particle size distribution profiling (Suppl. Figure 3E). NTA revealed a primary size distribution range of 50 nm to 150 nm, with a mean particle diameter of *d* = 82.7 nm. The distribution exhibited considerable variability, with a standard deviation of SD = 45.3 nm.

### Particle size distribution analysis of total EVs and L1CAM-EVs from Parkinson’s disease patients compared to control individuals

MISEV guidelines [9] emphasize the importance of identifying the complete particle size distribution in comparative studies. Figure 3 presents a detailed depiction of size distributions for plasma-derived EVs and L1CAM-EVs as visualized via TEM. For EVs, we analysed three images per individual in both the PD and HC cohorts (*n* = 5). Due to the lower particle density in L1CAM-EVs, we examined five images per individual (*n* = 3) in the same groups. The majority of EVs displayed diameters within the 20 nm to 50 nm range (*n*_*total*_*=*3082 particles; *d*_*mean*_*=*35.91 nm; *SD* = 10.41 nm) (Fig. 3A). The size distribution approximates a Gaussian curve within a two-standard deviation interval (2SD), though the peak is slightly right-shifted, indicating a mode approximately 4 to 5 nm below the overall mean (Fig. 3B). Notably, in the PD group, we observed a greater proportion of particles exceeding 60 nm in diameter compared to the HC group. For L1CAM-EVs, a similar analysis was performed. The resulting size distribution (Fig. 3C) reflects the pattern observed for EVs but with reduced overall total particle count (*n*_*total*_*=*923 particles; *d*_*mean*_*=*37.21 nm; *SD* = 8.11 nm). The size distribution of L1CAM-EVs (Fig. 3D) is notably less uniform than that of total EVs; however, a similar rightward peak shift is observed, consistent with the trend seen for total EVs.

In Fig. 3E, mean particle diameters of EVs and L1CAM-EVs are compared between PD and HC groups. For EVs, the mean diameter was significantly smaller in the HC group (*d*_*mean*_*=*35.44 nm) compared to the PD group (*d*_*mean*_*=*36.42 nm) (*p* = 0.0088). In contrast, for L1CAM-EVs, an inverse trend was observed: the HC group exhibited significantly larger mean diameters (*d*_*mean*_*=*38.38 nm) compared to the PD group (*d*_*mean*_*=*35.22 nm) (*p* ≤ 0.0001).

As illustrated in Fig. 3B and D, and consistent with the MISEV guidelines [9], the overall size distribution significantly impacts the calculated mean diameters, particularly in groups with a higher proportion of particles exceeding 80 nm. Notably, Such larger particles were rare, representing less than 5% of the total population in our analysis, and their presence appears to be sporadic. To minimize the effect of these outliers, a refined analysis is presented in Fig. 3F, focusing on particles with diameters in the range of 20–50 nm. This range corresponds to the calculated mean diameter ± approximately 2SD, thus encompassing over 95% of the particle population. Notably, particles smaller than 20 nm were almost entirely absent. Interestingly, this refined analysis revealed significantly increased particle diameters in the HC group for both, total EVs (*p* = 0.0005) and L1CAM-EVs (*p* = 0.0004).

### Algorithm-assisted analysis of TEM images

**Fig. 4:**
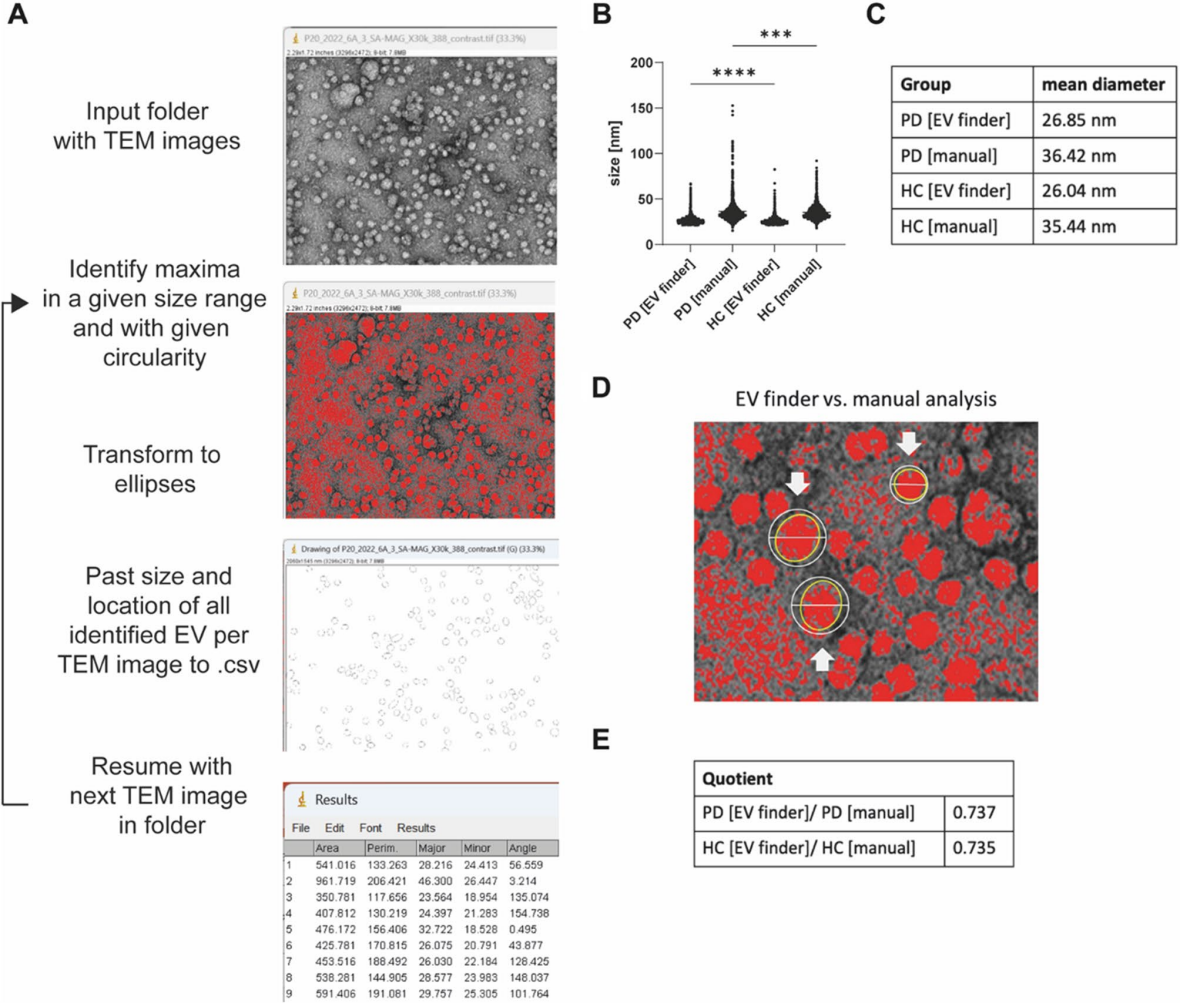
*EV finder*: semi-automated ImageJ script to analyse TEM images and quantify EVs. **A**) Schematic overview and exemplary data flow (for details see suppl. data). After input of a folder containing TEM images, the script will identify maxima that in size and circularity represent EV particles. The identified areas will be transformed into ellipses and the area and location of each ellipse will be stored in a CVS.txt file. All images from the same folder will be analysed in a loop like manner. CVS like.txt files can then be opened with other programs for subsequent analyses. **B** Comparative analysis of serum derived EV using manual and EV finder-based analysis. Statistical analysis using One-way ANOVA with *** *p* < 0.001 and **** *p* < 0.0001. **C** Quantitative comparison of the mean diameter for the different groups and analysis tools. **D** Comparison of EV finder areas (red and yellow ellipses) and manually added circles and diameters (white circles and arrows). **E** Quotient between mean particle diameter from manual and EV finder determined EV mean diameter size

Manual size measurements of EVs on TEM images is time-consuming, might not be exhaustive and prone to user-induced bias. Especially in terms of reproducibility, user experience is vital. To obtain an unbiased and exhaustive data set (measure all EVs on a TEM image), algorithms operating with the same threshold values might be beneficial. To facilitate rapid and reproducible TEM image analysis for EV size quantification, we developed an ImageJ-based plugin, which we named *EV finder*. *EV finder* makes use of ImageJ implemented tools and combines them in a single plugin (Fig. 4A). For this, TEM images are opened in ImageJ and a maximum search is conducted. For each data set individual threshold values have to be determined in terms of minimum/maximum size and minimum circularity (detailed description in suppl. Data: commented version of the *EV finder* (Version 1)). The areas are transformed into ellipses and the area and location of each individual ellipse is stored into a CSV style text (.txt) file (Fig. 4A). *EV finder* is designed to work through all images within a folder. As we had a manually analysed data set from PD patient and HC serum samples (Fig. 3), we reanalysed these images with the *EV finder*. Once all input values are set – a process that only takes a short time - the entire image analysis is completed within minutes. This is a significant improvement, as manual analysis takes hours or even days. For PD samples, *EV finder* identified 2,983 and for the control sample 4,915 particles as EVs in TEM images. When comparing the mean diameters of EVs determined manually with those measured using *EV finder*, it became clear that the diameters obtained with *EV finder* are significantly smaller than those obtained through manual analysis (Fig. 4B). Comparison of the absolute values exhibits a mean diameter for PD EVs from *EV finder* of 26.85 nm (SD = 4.97 nm) and for PD manual of 36.42 nm (SD = 12.36 nm). For HC, the sizes are 26.04 nm (SD = 4.23 nm) for *EV finder* and 35.44 nm (SD = 8.1 nm) for manually analysed EVs (Fig. 4C). When we examined the regions detected by the maximum finder in detail and compared them to manually measured diameters or areas, we identified a systematic error (Fig. 4D). Specifically, *EV finder* did not accurately detect the edges of EV particles. This occurred because the increasingly darker edges of the particles are no longer recognized as part of the maximum, leading to incomplete or underestimated particle measurements. However, this challenge highlights a known limitation of maximum-finding algorithms in image analysis: these methods typically identify points of highest local intensity, which may not correspond to the true boundaries of objects, especially when object edges are defined by gradual intensity transitions or darker regions. As a result, portions of particles with darker peripheries are omitted from detection, causing consistent underestimation of size compared to manual measurements.

When we calculated the ratio of manually measured EV mean diameters to those obtained with *EV finder*, we found nearly identical values for both groups (PD: 0.737; control: 0.735, Fig. 4E). Thus, despite considerable differences in absolute diameter measurements between *EV finder* and manual analysis, we believe that *EV finder* is a valuable tool that should be made available to the EV research community.

## Discussion

In this pilot study, we examined serum- and plasma-derived EVs from Parkinson’s disease (PD) patients and healthy controls (HC) by extensive manual TEM analysis and a newly developed ImageJ plugin for (semi-)automated EV diameter measurement (*EV finder*). Our data indicate that, although absolute size estimates differed depending on the method used, the relative changes between groups remained consistent. Notably, our findings suggest size differences in PD-derived total EVs as well as in L1CAM-positive EV fractions compared to HC. Future studies should validate our findings in larger, longitudinal cohorts and also include early-stage PD to clarify whether EV size differences persist throughout disease progression.

Precipitation reagents for EV enrichment are widely used in clinical settings for their speed and simplicity, though they yield lower purity compared to ultracentrifugation (UC) and size exclusion chromatography (SEC), which are more suitable for applications that require high-purity EVs [9, 12, 25, 50–53]. For TEM-based studies, precipitation methods have proven to be viable alternatives to UC or SEC. Especially co-enrichment of lipoproteins is a regular bias for precipitation-based methods. However, our samples were positive for the EV marker CD63 and metabolomics identified phosphatidylserine, which is only a minor component of lipoproteins but can be majorly found in EV. The lipid-rich profile is consistent with plasma- and brain-derived EV studies [45, 46]. L1CAM-EVs are currently the most commonly used target to isolate putative CNS-derived EVs [27, 29, 30]. However, the use of L1CAM as a marker for CNS-derived EVs has become increasingly controversial. Multiple studies have shown that L1CAM detected in blood and CSF is often present as a soluble protein rather than being EV-associated, likely due to proteolytic cleavage of its ectodomain and expression in non-neuronal tissues [20, 54]. This raises concerns about the specificity of L1CAM-based EV isolation, as soluble L1CAM fragments may bind nonspecifically to EVs or be co-isolated, potentially confounding results [20, 54]. Nonetheless, recent single-EV analyses and immunoelectron microscopy have demonstrated L1CAM co-expression with neuronal markers on a subset of EVs, suggesting that, when combined with additional markers, L1CAM can still provide useful enrichment for EVs [54]. Our finding that L1CAM-EVs constitute approximately 1% of total blood EVs is consistent with previous reports [20] and highlights the importance of using complementary markers and standardized protocols to improve specificity for CNS-EV isolation.

TEM imaging in this study revealed particle morphologies and densities consistent with published data [13, 40] as well as higher particle density of serum-EVs compared to plasma-EV populations. This might be explained by previous observations detecting increased platelet-derived EVs in serum [55–57]. The proportion of “cup-shaped” particles in our analyses (overall in all subgroups: ~5.3%) was lower than in a previous EV studies [14], and higher particle density correlated with fewer “cup-shaped” particles, supporting previous hypotheses [14].

Consistent with previous reports, NTA measurements yielded larger particle diameters than negative stain TEM due to the preservation of the hydration shell in NTA and its loss during TEM preparation [4, 9, 13, 15, 48, 50, 58, 59]. NTA may also under-detect smaller particles (< 60 nm), complicating direct comparisons. No significant morphological differences were observed between serum- and plasma-derived EVs, though pre-analytical variables such as collection tubes and anticoagulants can introduce variability and should be evaluated in future studies.

In both groups, PD patients and HC, particle size distributions were largely homogeneous. However, when focusing on the most frequently observed size range (20–50 nm), the PD group exhibited significantly smaller mean diameters for both EVs and L1CAM-EVs compared to the HC group, as seen in a previous study [40]. Most studies have focused on biochemical markers such as α-synuclein for distinguishing PD patients from HC and other synucleinopathies [28, 29, 35, 37, 60–62]. Hence, combinatorial approaches (e.g., integrating NTA or TEM with α-synuclein quantification) may enhance diagnostic potential as it increases separation precision.

EVs show growing promise for improving diagnostics and therapeutics, especially in neurological diseases, but differences in isolation and analysis methods remain a major challenge. Our study demonstrates the practical advantages of precipitation-based isolation and highlights the potential of TEM imaging—particularly when paired with automated analysis (e.g. by using our *EV finder*) —in providing reliable particle characterization. We advocate for broader use and further automation of TEM analysis in EV research, as integrating this approach with biochemical and NTA data could substantially advance the development of consistent protocols and accurate diagnostic markers.

## Supplementary Information

Supplementary Material 1.

Supplementary Material 2.

## Data Availability

All metabolomics data is provided within the supplementary material and will be provided upon reasonable request.
