# Supplementary material for: Transmission Electron Microscopy-based characterization of Extracellular Vesicles from plasma and serum from Parkinson´s Disease patients
Source: Cell Commun Signal. 2025 Sep 18;23:395. doi: 10.1186/s12964-025-02383-w (PMC12445024; doi:10.1186/s12964-025-02383-w)
Supplement: Supplementary file 2 — Supplementary Material 2. [file 12964_2025_2383_MOESM2_ESM.pdf]

Supplemental Data

Supplemental Figures

A

	Donor abbr.	Collection date	Age (years)	Sex	Height (cm)	Weight (kg)	Last meal (h b. coll.)
PD	PD1	03/14/2022	64	female	162	82	14
	PD2	02/28/2022	71	female	157	63	4
	PD3	03/07/2022	63	female	160	105	4
	PD4	03/14/2022	54	female	175	75	5
	PD5	05/23/2022	67	female	158	72	1
HC	HC1	12/21/2021	68	female	151	54	4
	HC2	12/03/2021	50	female	160	50	2.5
	HC3	12/17/2021	72	female	n.r.	n.r.	3
	HC4	12/16/2021	59	female	180	117	3
	HC5	13/03/2021	66	female	162	68	2.5

	Donor abbr.	Diagnosis subtype	Duration of disease (years)	UPRDS	Hoehn & Yahr stage	Daily L-Dopa dose (mg)
PD	PD1	tremor-dominant type	8	11	2	1221
	PD2	akinetic-rigid type	12	24	3	1920
	PD3	akinetic-rigid type	12	15	2	850
	PD4	equivalence type	9	30	2	650
	PD5	akinetic-rigid type	18	33	4	1149

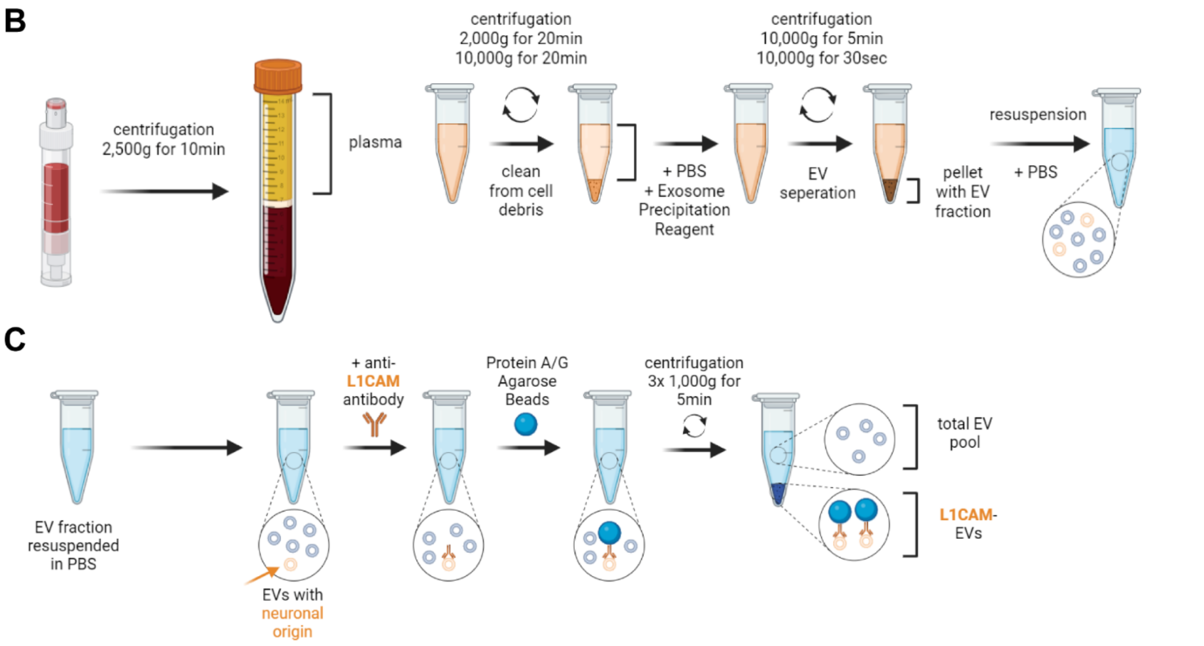

Suppl. Figure 1: Protocol, Demographics. A. Demographic overview over PD patients and HC individuals, included in our analysis. . **B.** Schematic overview of the EV isolation protocol, performed with ThermoFisher´s Total Exosome Isolation Kit **A+B** are created with biorender.com. **C.** Immunoprecipitation protocol for L1CAM-EVs

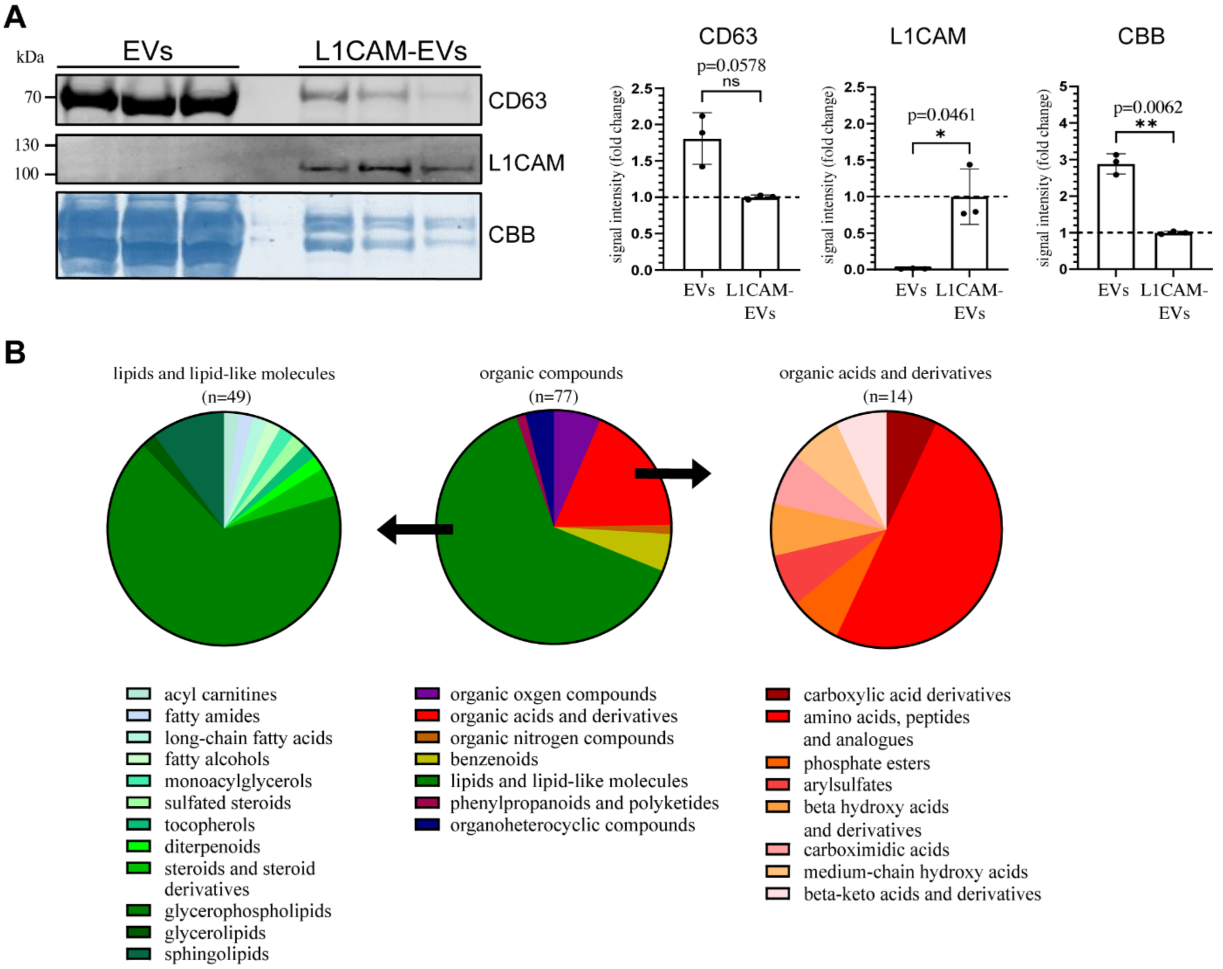

Suppl. Figure 2: Biochemical Characterization of EVs and L1CAM-EVs. A. Western blot analysis of EVs and L1CAM-EVs, isolated from plasma of *n=3* HC individuals, stained for CD63, L1CAM and CBB (Coomassie Brilliant Blue). Bar graphs indicate changes, with CD63 and L1CAM-signals normalized to total protein amount detected through CBB staining. Mean L1CAM-EV signals were established as the baseline with a numerical representation of 1. Error bars show mean values with SD. Ns = not significant, */** = significant with *p*-value <0.05, *p*-values are provided in each bar graph, unpaired t-test with Welsh´s correction was used to determine statistical significance. **B.** Metabolomic enrichment analysis of *n*=3 plasma-derived EV samples of one HC individual. Middle circular diagram represents all detected compounds, divided by biochemical classes and subclasses of the molecules. The left chart illustrates more detailed the composition of all "lipids and lipid-like molecules", the right chart provides information about the molecules present in the group "organic acids and derivatives".

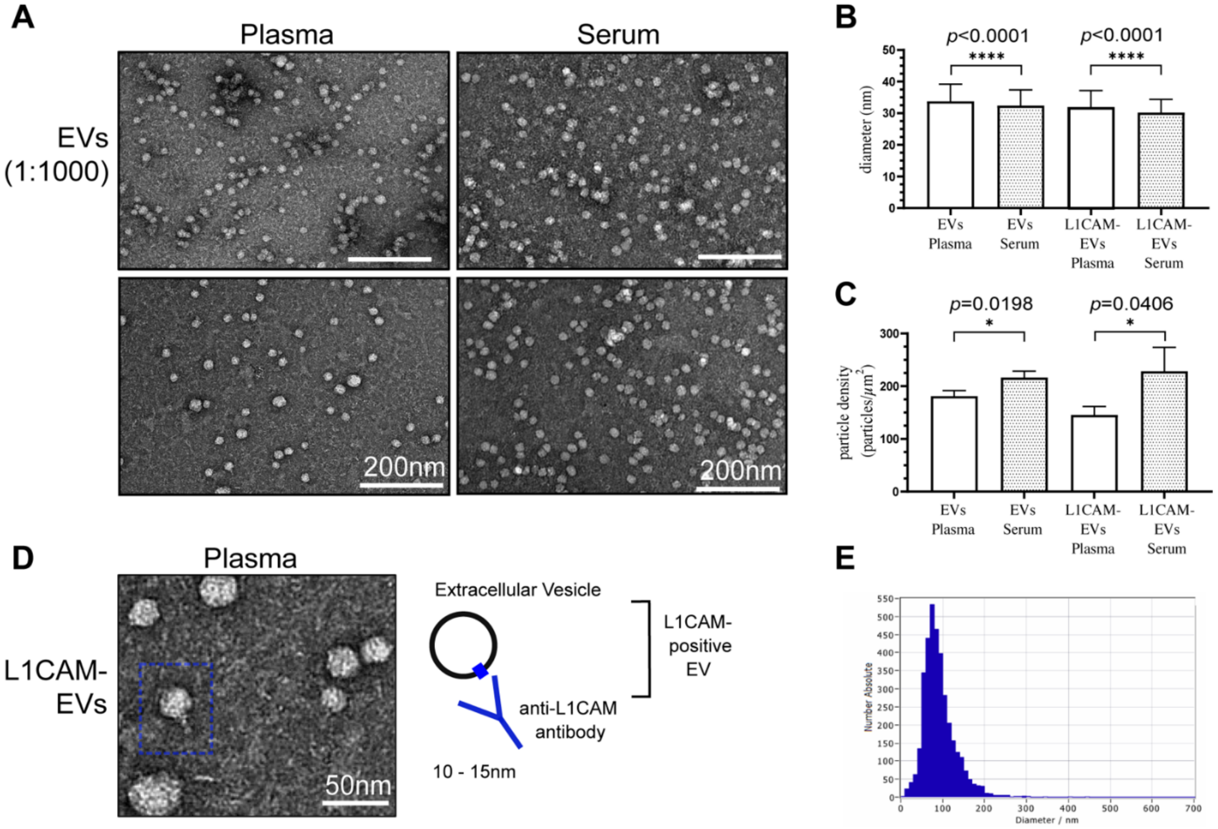

Suppl. Figure 3: Comparison Plasma / Serum. A. Exemplary images of *n=1* HC individual of EVs and L1CAM-EVs isolated from plasma and serum. EVs are diluted in 0.9% NaCl 1:100. Scale bar = 200nm. **B.** Diameter comparison between EVs and L1CAM-EVs isolated from plasma and serum, by measuring *n*=100 particles in each image. **C.** Density comparison between plasma- and serum-derived EVs and L1CAM-EVs, measuring the detected particles in a defined frame, calculated to particles per square µm. **B+C.** Error bars show mean values ± SD, unpaired t-test was performed to determine statistical significance. * / **** = significant with $p<0.05$. **D.** Enlarged area from **A** (blue box from L1CAM-EVs Plasma), showing particle binding to presumably anti-L1CAM antibody. Scale bar = 50nm. The assumed interaction is schematically shown on the right side of the image. **E.** NTA results (ZetaView) from plasma-derived EVs, isolated from one HC individual, showing the distribution of particles, with the particle diameter along the x-axis and the number of particles with a certain diameter along the y-axis. To determine mean values and SD, we evaluated *n*=3 measurements in number-weighted distribution of the ZetaView program.

Suppl. Table 1

Antibody target	Host	Company	Ref. number	Dilution
CD63 LEAF™	rabbit	BioLegend, San Diego, CA, United States	353013	1:1,000
L1CAM C2 (NCAM-L1 / CD171)	mouse	Santa Cruz Biotechnology, Dallas, TX, United States	sc-551430	1:500
Anti-rabbit Alexa Fluor 680	donkey	ThermoFisher Scientific Inc., Waltham, MA, United States	A10043	1:10,000
anti-mouse IRDye® 800CW	donkey	LI-COR Biosciences, Lincoln, NE, United States	926-32212	1:10,000

Commented version of the *EV finder* (Version 1)

To gain a better understanding of the macro, this serves as a commented version. The comments are added above the respective line of code, with // at the beginning of every comment.

// processFolder is a function that processes a folder of images. First it creates a list of the files, then loops over the list, finds files with the required datatype and calls the processImage function for every image. Subfolders are also processed by calling the processFolder function again for each subfolder. If there are any images of another datatype encountered, they are skipped with "continue"

```
function processFolder (directory) {
	// get the list of files in it
	filelist = getFileList(directory);

	// go over the list
	for (i = 0; i < lengthOf(filelist);i++) {
		// only collect images of the selected datatype
		If (endsWith(filelist[i], datatype)){ // endsWith(string,suffix)
			processImage(directory,filelist[i]);
			resultCounter++;
		} else if (endsWith(filelist[i], "/")){
			// is a subfolder
			processFolder(directory + filelist[i]);
		} else {
		continue;
		}
	}
}
```

// function where the steps of the analysis happen. Opens the image, converts to 8-bit, enhances the contrast, applies the measurements, sets a threshold of the chosen thresholding method, sets the scale and runs analyze particles with input parameters. Saves an image with ellipses of all analyzed EVs and a table with the measurement results

```
function processImage (directory, filename) {
	// open the image
	open(directory + filename) ;
	// convert to 8 bit if necessary, .tif files are usually in 16 bit
```

```
            run("8-bit");
            run("Set Measurements...", "area perimeter fit redirect=None decimal=3");
            run("Enhance Contrast...", "saturated=0.4");
            If (threshold == "automatic") {
                                setAutoThreshold("Default dark");
            } else if (threshold == "manual") {
                        setThreshold(lowerThreshold, upperThreshold);
                        setOption("BlackBackground", false);
            }
            run("Set Scale...", " distance=" + distanceInPixel +  " known=" + knownDistance+ " unit="+
            unitOfLength + " global");
            run("Analyze Particles...", "size=" + sizeFrom + "-" + sizeTo + " circularity=" +
            circularityFrom + "-" + circularityTo + "show=Ellipses clear include");
            // the next two lines save the Ellipses data with the same name as the image + "ellipses"
            ellipsesTitle = getTitleStripExtension();
            saveAs("TIFF", directory + ellipsesTitle + "_ellipses");
            close(); // closes active image (in this case the ellipses)
            // saves results as txt data, separate for each image, with the image name + result
            title = getTitleStripExtension();
            saveAs("results",directory+title+" result.txt");
}

// function that returns the title of an image without its extension. This is just a little function to get rid of the extension to easier name the results and ellipses.
function getTitleStripExtension() {
  t = getTitle();
  t = replace(t, ".tif", "");
  t = replace(t, ".tiff", "");
t = replace(t, ".bmp", "");
   return t;
}
```

// With the batch mode applied, all images that are displayed when using ImageJ manually, are hidden during macro execution. This enhancement results in an increase in the efficiency and speed of the macro.

```
setBatchMode(true);
```

// in the following there are different windows created to interact with the user. Some are just displaying important information; others are used to gather input from the user.

// this line generates a window where the user can select a folder to analyze, by using the getDirectory function. This is an already implemented function of the ImageJ Macro langugae, so it can be called directly.
An overview of those functions can be found here: https://wsr.imagej.net/developer/macro/functions.html

```
directory = getDirectory("Choose a folder of TEM images (.tif) you want to analyze");
```

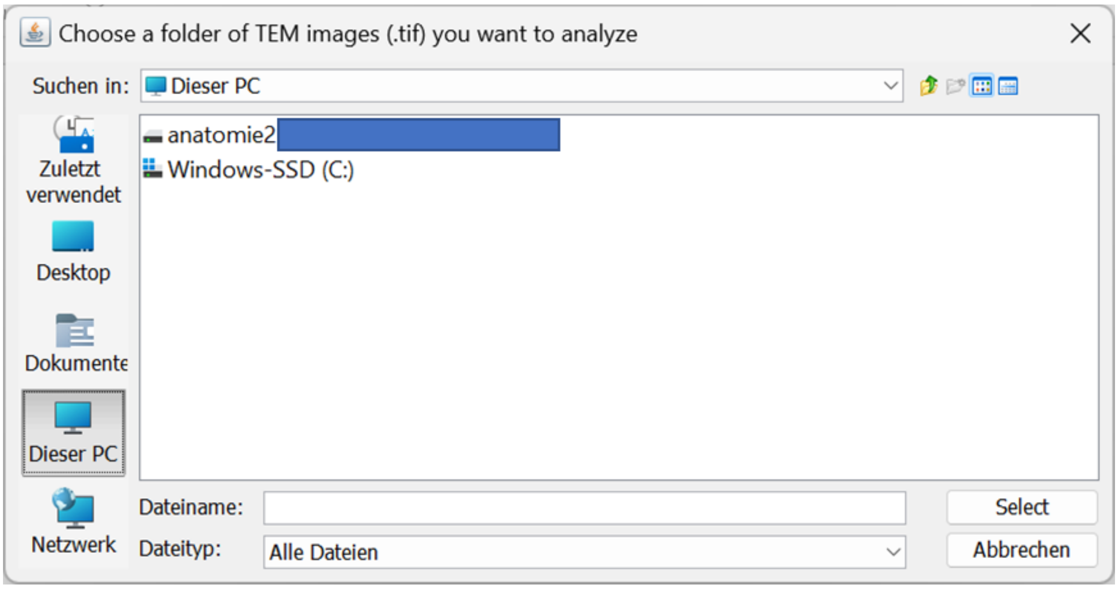

// the following lines create a window to select which datatype needs to be analyzed (.tif or .bmp)

```
datatypeArray = newArray(".tif",".bmp");

Dialog.create("Datatype");

Dialog.addChoice("Choose which datatype to analyze", datatypeArray);

Dialog.show();
```

// after the user clicked ok, the selected choice is stored in the datatype variable

```
datatype = Dialog.getChoice();
```

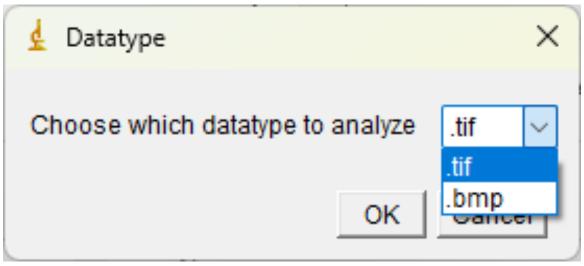

// the next two lines display a window to explain, that the results are stored in the same folder as the input data. It also gives information about which measurements are taken. This can be easily done using the pre-implemented Dialog functions.

```
Dialog.create("Results and Measurements");

Dialog.addMessage("The results are stored in the same folder as your input images");

Dialog.addMessage("This macro measures the area, the perimeter and the two axis of a best fitting ellipse to every particle");

Dialog.show();
```

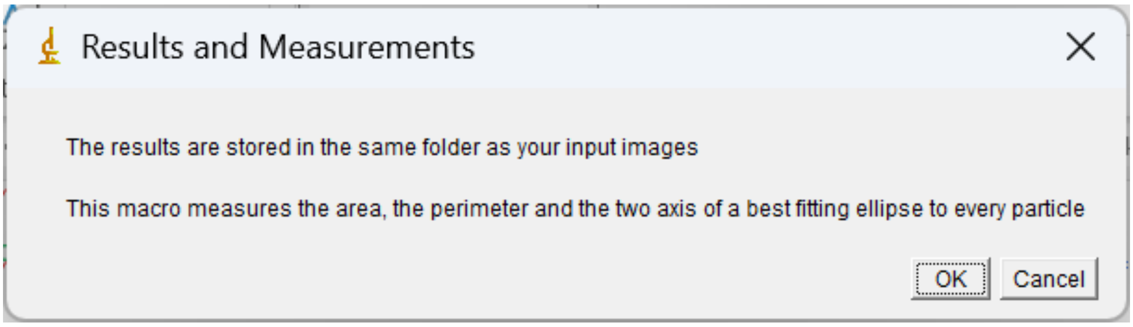

// produces a window to choose between manual and automatic thresholding by the use of a dropdown menu

```
threshold = "threshold";

thresholdArray = newArray("automatic","manual");

Dialog.create("Thresholding method");

Dialog.addChoice("Choose which thresholding method you want to use ", thresholdArray);

Dialog.show();
```

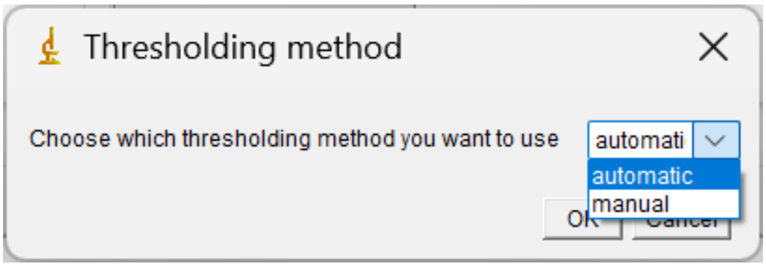

// return the chosen option, save it in the threshold variable for later use

```
threshold = Dialog.getChoice();
```

// the following code is only executed if the user selected the manual thresholding method. The automatic thresholding is implemented with fewer code and is executed in the processImage function below.

However, when choosing the manual thresholding there are some additional steps necessary. First there is a window created which asks for the upper and lower threshold, and those inputs are then stored in the corresponding variable. To know the thresholds that works best for your image, some manual testing is required before (specific steps are defined in the instructions).

```
if (threshold == "manual") {

    // Setting the threshold, default values

    lowerThreshold = 100;

    upperThreshold = 255;

    // creates the dialogbox for threshold settings

    Dialog.create("Manual threshold settings");

    Dialog.addMessage("Please enter the upper and lower threshold you want to apply");

    Dialog.addNumber("lower threshold", lowerThreshold);

    Dialog.addNumber("upper Threshold", upperThreshold);

    Dialog.show();
```

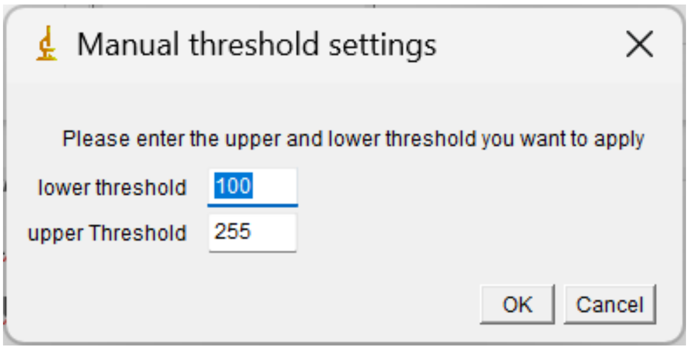


```
    // receives and stores input in corresponding variables

    // the values are recovered in order of appearance, that's why the following two lines need to be in this specific order and can't be turned around.


    lowerThreshold = Dialog.getNumber();

    upperThreshold = Dialog.getNumber();

}
```

// in the next section the variables for the Scale Settings are created and initialized with default values

```
distanceInPixel = 338;

knownDistance = 200;

pixelAspectRatio = 1.0;

unitOfLength = "nm";
```

// Note: the pixel aspect ratio is not displayed, so that it can't be changed by accident. The "Fit Ellipse" selection is used in measurements, which is only working with the pixel aspect ratio set to 1.0. Having a pixel aspect ratio of 1.0 means that the pixel themselves are squares. ImageJ displays pixel as squares, but other imaging devices might use rectangular pixels.

// The next code creates the Dialogbox for Scale Settings. In this window the user can modify the measurements that are taken. Without these settings, the results are automatically created in pixel and pixel^2. To convert those into the chosen unit, there are a few variables where the input is stored and then later be used in "run(Set Scale…)

```
Dialog.create("Scale Settings");

Dialog.addMessage("Please enter the measurements of your images to receive output data in your chosen unit");

Dialog.addNumber("Distance in pixels", distanceInPixel);

Dialog.addNumber("Known Distance", knownDistance);

Dialog.addString("unit of length", unitOfLength);

Dialog.show();
```

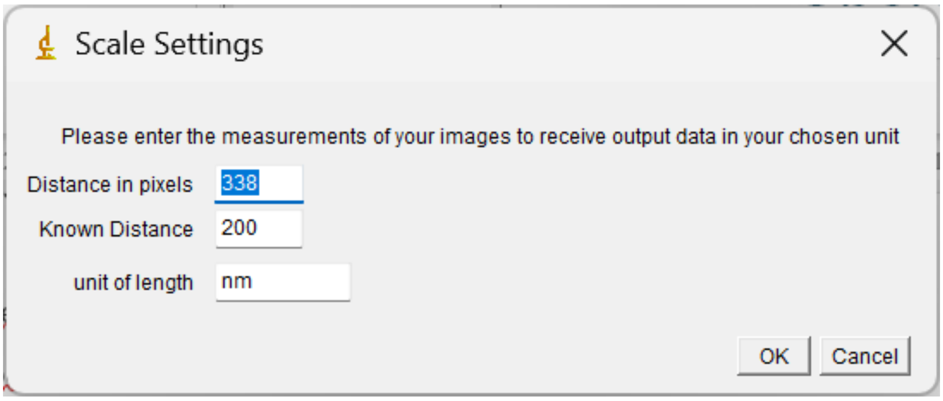

// receiving and storing input in corresponding variables

```
distanceInPixel = Dialog.getNumber();

knownDistance = Dialog.getNumber();

unitOfLength = Dialog.getString();
```

// In the following code, the variables for Analyze Particles are generated and initialized. There are two variables required for size and circularity to later be combined to serve as a range.

```
sizeFrom = 350;

sizeTo = 11400;
```

circularityFrom = 0.15;

circularityTo = 1.00;

// The code creates a dialogbox for Analyze Particles where different input data is needed, and default values are suggested. For the circularity the input format is very important. ImageJ itself has a default value in the following format displayed: "x.xx". Experiments where it was changed to "x,xx" concluded in significantly different results. Therefore, it is important to display this info to the user, as the program runs with the wrong format and does not give back any feedbackor error.

```
Dialog.create("Analyze Particles");

Dialog.addMessage("Please enter the parameters to determine which particles to analyze");

Dialog.addNumber("size from", sizeFrom); // (displayed name, default)

Dialog.addNumber("to ", sizeTo);

Dialog.addMessage("Circularity must be in the following format for decimal numbers! x.xxx");

Dialog.addMessage("if you use x,xx your output data is wrong");

Dialog.addNumber("circularity from", circularityFrom);

Dialog.addNumber("to ", circularityTo);

Dialog.show();
```

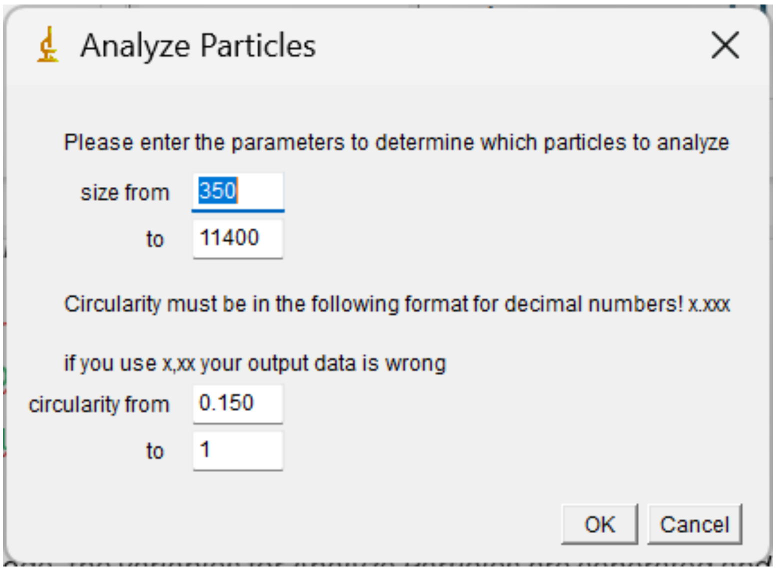

// code execution after user clicked ok, saves inputs into variables in the order they are displayed (top to bottom)

```
sizeFrom = Dialog.getNumber();

sizeTo = Dialog.getNumber();

circularityFrom = Dialog.getNumber();

circularityTo = Dialog.getNumber();
```

// Displays window to not close the programm until told because it takes some time

```
Dialog.create("Start");

Dialog.addMessage("When you click ok, the program starts.");

Dialog.addMessage("Please don't close ImageJ or interrupt the program until it's done");

Dialog.show();
```

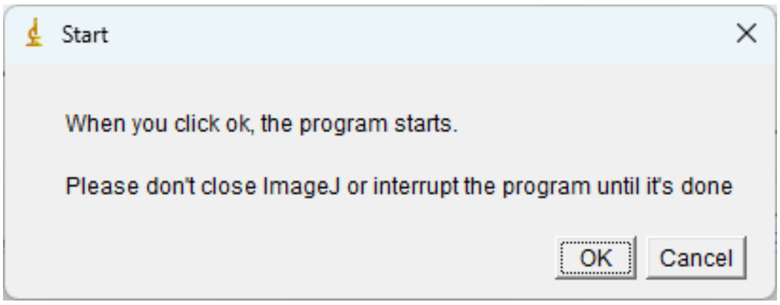

// resultCounter gives back how many images were analyzed. +1 is added to the counter each time an image is analyzed. At the end the current number saved in the counter is displayed to the user. This is an important feedback tool to see if all images were analyzed or if e.g. zero images were analyzed because something went wrong in the progress. Ideally the user would then check the instructions again or consult the trouble shooting guide.

```
resultCounter = 0;
```

// here the function processFolder is called with the folder the user selected at the beginning of the programm

```
processFolder(directory);
```

// the following two lines are two important feedback lines provided through the log window of ImageJ

```
print(resultCounter + " images were analyzed");

print("All images were analyzed, you can now close the programm");
```
